# Long-Chain Polyunsaturated Fatty Acid Status at Birth and Development of Childhood Allergy: A Systematic Review

**DOI:** 10.3390/life12040526

**Published:** 2022-04-02

**Authors:** Tamás Decsi, Tamás Marosvölgyi, Eszter Muszil, Blanka Bódy, Éva Szabó

**Affiliations:** 1 Department of Pediatrics, Clinical Centre, University of Pécs, 7623 Pécs, Hungary; decsi.tamas@pte.hu (T.D.); muszileszter@gmail.com (E.M.); body7991@gmail.com (B.B.); 2 Institute of Bioanalysis, Medical School, University of Pécs, 7624 Pécs, Hungary; marosvolgyi.tamas@pte.hu; 3 Department of Biochemistry and Medical Chemistry, Medical School, University of Pécs, 7624 Pécs, Hungary

**Keywords:** allergy, arachidonic acid, atopy, children, cord blood, docosahexaenoic acid, eicosapentaenoic acid, eczema, infant, long-chain polyunsaturated fatty acid

## Abstract

The associations of fetal fatty acids status to immune-related health parameters later in life are unclear. Our aim is to collect all available information on the relationship between fatty acid status at birth and allergy in childhood. Systematic literature search was performed on Ovid MEDLINE, Cochrane Library, and Embase. The search retrieved 897 articles without duplicates; 14 articles remained after excluding those that did not fit into our inclusion criteria. When the dichotomous parameter of suffering or not from allergic condition in childhood was analyzed, cord blood eicosapentaenoic acid (EPA) values proved to be significantly lower in allergic than non-allergic children in four comparisons from three studies. When the linear parameters of odds ratios and relative risks for allergy were taken into consideration, high cord blood EPA, but also high docosahexaenoic acid (DHA) and high total n-3 long-chain polyunsaturated fatty acid values were associated to clinically relevant reduction (at least 38%) in eight comparisons from five studies. Within the cord blood samples, higher EPA, docosapentaenoic acid, and DHA values were significantly and negatively associated in eight correlation analyses from three studies with laboratory parameters considered to reflect allergic trait. The data reported here may provide information for defining optimal fatty acid intakes for pregnant women.

## 1. Introduction

The most important long-chain polyunsaturated fatty acids (LCPUFAs), docosahexaenoic acid (C22:6n-3, DHA) and arachidonic acid (C20:4-6, AA), are bioconversion products from the essential fatty acids (EFAs), alpha-linolenic acid (C18:3n-3, ALA) and linoleic acid (C18:2n-6, LA), respectively. Although the efficiency of the conversion of EFAs to LCPUFAs is questioned throughout the human lifespan [1], it can be assumed with good reason that the perinatal period of rapid human growth and development represents the most critical stage. The need of LCPUFA supply to the human fetus was emphatically put forward as early as nearly half a century ago [2], and five decades of ongoing research yielded extensive data in this field.

Overall, 70 randomized controlled trials (RCTs) involving 19,927 pregnant women and investigating the addition of n-3 LCPUFAs either as supplements or as foods to the diet were summarized in a relatively recent Cochrane review [3]. Both preterm birth [< 37 weeks; relative risk (RR): 0.89; 95% confidence interval (95% CI): 0.81 to 0.97; 10,340 participants] and early preterm birth [<34 weeks; RR: 0.58; 95% CI: 0.44 to 0.77; 5204 participants] were significantly lower in women who received n-3 LCPUFA compared with no n-3 fatty acids. There was also a possibly reduced risk of perinatal death (RR: 0.75; 95% CI: 0.54 to 1.03; 7416 participants) and possibly fewer neonatal care admissions (RR: 0.92; 95% CI: 0.83 to 1.03; 6921 participants). In parallel with reduction in preterm birth and early preterm birth, prolonged gestation (>42 weeks) increased in women who received n-3 LCPUFA compared with no n-3 fatty acids (RR: 1.61; 95% CI: 1.11 to 2.33; 5141 participants). Important conclusions of this Cochrane review were that: (a) more studies comparing n-3 LCPUFA and placebo to establish causality in relation to preterm birth are not needed, and (b) further trials are needed to improve understanding of underlying mechanisms [3].

Development of the immune system is one of the major mechanisms influencing fetal wellbeing and pregnancy outcome, and patterns of LCPUFA exposure in pregnancy was reported to influence the fetal immune system. Decreased consumption of n-6 EFA and LCPUFAs in favor of more anti-inflammatory n-3 EFA and LCPUFAs in modern diets, has demonstrated the potential protective role of n-3 fatty acids in allergic and respiratory diseases [4,5,6]. Moreover, n-3 EFA and LCPUFAs may alter the T helper cell balance by inhibiting cytokine production and may further modify cellular membrane and induce eicosanoid metabolism and later gene expression [4,7,8].

Human studies showed that higher levels of n-3 LCPUFA were associated with reduction in neonatal oxidative stress, reduced production of inflammatory leukotriene B4, and altered T-cell function [9,10]. Inverse correlations between n-3 LCPUFA levels and neonatal T-cell cytokine production were also reported, consistent with data of adult studies showing reduction of T-cell cytokine production with fish oil supplementation [9,11]. Moreover, intrauterine LCPUFA supply may be associated with beneficial immunological consequences also after birth. Studies on supplemental n-3 LCPUFAs in pregnant women demonstrated reduced sensitization of infants to egg, reduced risk and severity of atopic dermatitis in the first year of life, and reduced persistent wheeze and asthma at ages of three to five years [12]. These observations indicate that LCPUFAs play a clinically significant role in immune development.

Despite encouraging data of the beneficial effects of LCPUFA supplementation to pregnant women on long-term immunity in the offspring, the exact associations of fetal fatty acids status to immune-related health parameters later in life are yet to be determined. Therefore, we decided to carry out a systematic review on objectively assessed neonatal fatty acids status at birth and occurrence of allergic diseases and clinical signs of atopy in early childhood.

## 2. Materials and Methods

This systematic review was registered prospectively in PROSPERO, under: CRD42021281397. The methodology and the results are reported according to the PRISMA (Preferred Reporting Items for Systematic Reviews and Meta-Analyses) guidelines for systematic reviews [13,14] (Table S1), as well as the guideline of the Cochrane Handbook of Systematic Reviews and Intervention [15].

### 2.1. Eligibility Criteria

We included studies on biological samples, allowing evaluation of LCPUFA status of healthy term newborns at birth (cord blood lipids, cord vessels wall lipids); whereas fatty acid studies on maternal biological samples obtained at delivery, on human milk (colostrum), or those on biological samples obtained from the offspring after birth, as well as data of not healthy infants (including preterm babies) were excluded. We included studies on fatty acid status but excluded studies on other blood lipids (e.g., HDL, LDL cholesterol, and triglyceride). Studies on incidence and prevalence of allergies, clinical signs of atopy, or other immune-related soluble factors in childhood were included, but inflammation in general or inflammatory factors were excluded. No restriction on clinical study type (observational or RCT) was applied; we excluded reviews, editorials, and comments not publishing original data. To sum up, we included studies reporting on fatty acid status in healthy newborns at birth in correlation with allergy-related data obtained in infancy or childhood.

### 2.2. Literature Search

Electronic literature search was performed on the following databases from the inception of each database up to September 2021: Embase, Cochrane Central Register of Controlled Trials (CENTRAL), and Ovid MEDLINE. No language restriction was applied. The search strategy was developed with terms related to newborns, allergy, and fatty acids. The search strategy on Ovid MEDLINE was as follows: (cord.mp OR newborn.mp OR infant.mp OR perinatal.mp OR postnatal.mp) AND (immune.mp OR immune*.mp OR allergy.mp OR allerg*.mp OR atopy.mp OR atopic.mp OR inflammation.mp OR infection.mp) AND (arachidonic.mp OR arachidonic acid.mp OR docosahexaenoic.mp OR docosahexenoic.mp OR docosahexaenoic acid.mp). The detailed search strategy for Ovid MEDLINE is available in List S1.

We manually searched the references of the included articles and related reviews for potentially relevant articles. We also searched grey literature for congress abstracts that might be relevant. All citations were then combined, and duplicates were excluded.

### 2.3. Study Selection, Risk of Bias Assessment

Pairs of review authors (B.B., E.M., É.S., T.M.) independently screened the abstract, title, or both of every record to determine potentially relevant articles. The abstract screening was performed on an online program (http://abstrackr.cebm.brown.edu; accessed on 15 November 2021) [16]. Then, the pairs of reviewers screened the full-text articles for inclusion and exclusion criteria independently on Rayyan.ai [17]. Disagreements between reviewers were resolved by further discussion until consensus. Risk of bias was also assessed independently by two authors (É.S., T.M.) according to the Cochrane Risk of Bias 2 (RoB2) tool for the RCTs [18], while the cohort studies and case-control studies were assessed using the ROBINS-I tool [19].

### 2.4. Data Extraction and Synthesis

One author (É.S) extracted data from the included articles, while the other author (T.M.) checked the data for accuracy and completeness. From full-text publications, the following data were extracted: first author, journal, year of publication, study type, place of study, type of sample, and reported outcomes. If the study published outcomes for multiple time points, we extracted data for each time point.

## 3. Results

**Figure 1:**
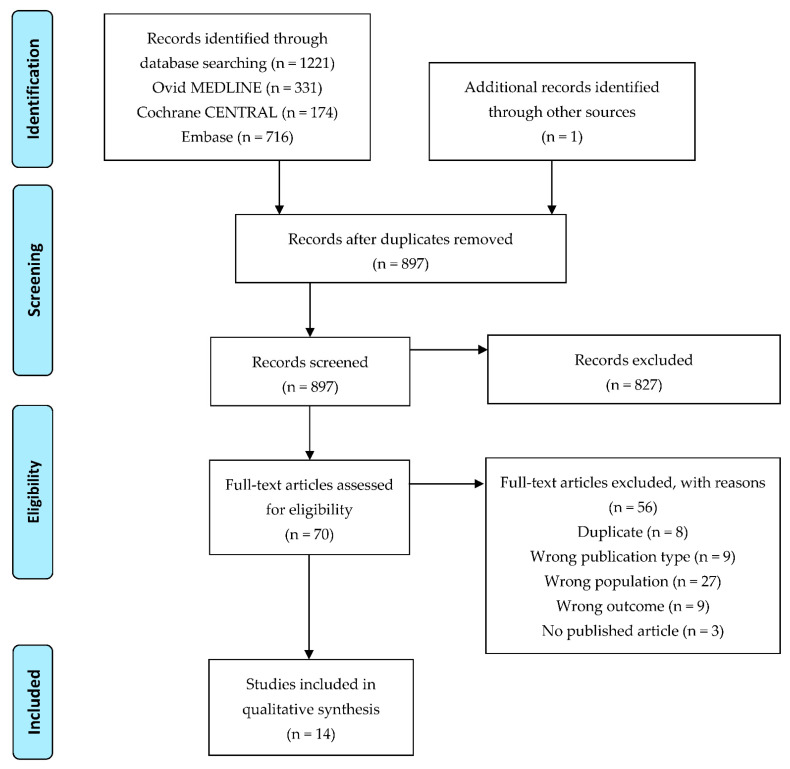
Flow diagram of study selection.

After removing duplicates, the search resulted in 897 articles (Figure 1). After title/abstract screening, most articles were excluded; full text screening was evaluated on 70 articles. From these articles, eight were duplicates of another publication with the same population and same time point [20,21,22,23,24,25,26] and nine were excluded because they were reviews [27,28,29,30,31] or in vitro cell studies (cultured cells from cord blood) [32,33,34,35]. Furthermore, 27 articles were excluded because they investigated wrong populations; in many studies, infants were supplemented [36,37,38,39,40,41,42,43,44,45,46,47], while in others, blood was drawn later than at birth [48,49,50,51,52,53,54,55,56,57]; one study investigated placental lipid fatty acid composition [58], in two other studies no blood was drawn from the child [59,60], and in two further studies breast milk fatty acid composition was studied [61,62]. In nine studies, the outcome was not within our inclusion criteria (there was no direct link between fatty acids and immune-related factors [63,64,65,66,67,68,69,70], or inflammatory markers were only studied [71]). We also found two conference abstracts [72,73] and a trial protocol [74] where results have been not published in full article yet. Finally, 14 articles were included in the analysis (Table 1).

### 3.1. Description of Included Studies

Most of the included studies were either RCTs (*n* = 6) [75,76,77,78,79,80] or birth cohort studies (*n* = 5) [81,82,83,84,85]. Three studies were published in two different articles [75,78,79,80,81,82], in that either the parameters analyzed in cord plasma [78,79] or the follow-up points [75,80,81,82] were different. For the purposes of the present review, we considered these related articles as independent publications.

Six studies reported cord blood fatty acid compositional data after maternal fish oil or n-3 LCPUFA supplementation during pregnancy, while four studies reported cord blood fatty acid data according to the appearance of allergy/atopy later on. Various cord blood constituents were used to characterize fatty acid status at birth: total plasma in four studies alone [78,79,84,86] and in one study in combination with umbilical artery and vein wall lipids [83], total serum in one study [87], plasma or serum phospholipids in five studies [76,77,81,82,88], and red blood cell (RBC) membrane lipids in three studies [75,80,85]. We provide detailed description of the type of lipid sample used, the method of fatty acid determination, and the list of fatty acids published in the given study in Table S2. Different surrogate parameters of allergy in the offspring were evaluated at birth in five studies [75,78,79,80]; we give description of the putative usefulness of these surrogate parameters in Table S3. Direct clinical signs and symptoms for allergy were estimated at no less than 11 different ages from 6 months [84] to 8 years [82].

When we compared the fatty acids identified and reported by the different studies (Table S2), we found that most of the articles presented fatty acid data as a percentage of total fatty acids by weight [76,79,80,81,82,83,84,85,87,88]. Only one study presented it as μg/10^6^ cells [75], one as μmol/L [86], and one as mol% [77]. As for fatty acid determination, most research groups used the generally accepted capillary gas chromatographic (GC) determination [75,76,80,83,87], mostly with flame-ionization detector (FID) [77,81,84,85,86], and only a few determined fatty acids by mass spectrometry (MS) [79,82,88].

Three articles published no information about the family history of allergies [78,79,81], in two articles some newborns had at least one allergic parent [82,83], and in one article the number of newborns with a family history of maternal allergy was not described, but data were controlled for this confounding factor in the multivariate analysis [85]. Only two studies included healthy mothers with no allergic history [84,86], while in one article the included mothers were divided into two subgroups based on allergy [87]. The other articles investigated newborns of allergic mothers [75,80] or high-risk newborns with a family history of allergic disease [76,77,88]. In the included studies, twelve had moderate risk of bias, while serious risk of bias was identified in two studies (Tables S4 and S5).

**Table 1:** Characteristics of the included studies.

First Author, Year of Publication	Study Type	Place of Study	Subgroup of Infants	Type of Sample	Investigated Immune-Related Diseases/Factors
Barden AE, 2004 [75]	RCT	Subiaco, Australia	Maternal fish oil or olive oil supplementation during pregnancy	Cord blood RBC	Cord plasma F_2_-isoprostanes
Barman M, 2019 [81]	Birth cohort study	Sweden	Mothers living on a farm/not	Cord blood serum PL	Allergy at 18 and 36 months
Barman M, 2020 [82]	Birth cohort study	Sweden	Mothers living on a farm/not	Cord blood serum PL	Allergy at 1, 3, 5, and 8 years
Best KP, 2018 [76]	RCT	Australia	Maternal n-3 LCPUFA or placebo supplementation	Cord blood plasma PL	Allergic symptoms at 1, 3, and 6 years
Byberg K, 2008 [86]	Nested case-control study	Stavanger, Norway	Atopic/non-atopic	Cord blood plasma	Atopy, sCD23, and IgE at 3 years
Dirix CEH, 2009 [83]	Birth cohort study	Maastricht, The Netherlands	No	Cord blood plasma PL; umbilical artery and vein walls PL	Immune-related measurements at 7 years
Furuhjelm C, 2011 [77]	RCT	Sweden	Maternal n-3 LCPUFA or placebo supplementation	Cord blood plasma PL	Allergic symptoms up to 2 years
Galli E, 1994 [88]	Cohort study	Rome, Italy	Atopic/non-atopic	Cord blood serum PL	Atopy in the first 12 months
Montes R, 2013 [84]	Birth cohort study	Sabadell, Spain	Atopic/non-atopic	Cord blood plasma	Atopic eczema at 6 and 14 months
Mozurkewich EL, 2016 [78]	RCT	Michigan, USA	Maternal EPA/DHA/placebo supplementation	Cord blood plasma	Specialized pro-resolving mediators in cord plasma
Mozurkewich EL, 2018 [79]	RCT	Michigan, USA	Maternal EPA/DHA/placebo supplementation	Cord blood plasma	Cytokines in cord plasma
Newson RB, 2004 [85]	Birth cohort study	UK	No	Cord blood RBC PL	Wheezing and eczema at 18–30 and 30–42 months
See VHL, 2017 [80]	RCT	Subiaco, Australia	Maternal fish oil or olive oil supplementation during pregnancy	Cord blood RBC	Pro-resolving mediators at birth and 12 years
Yu G, 1996 [87]	Case-control study	Linköping, Sweden	Allergy: yes/no	Cord blood serum PL	Allergy during the first 6 years

DHA: docosahexaenoic acid, EPA: eicosapentaenoic acid, LCPUFA: long-chain polyunsaturated fatty acid, PL: phospholipids, RBC: red blood cells, RCT: randomized controlled trial.

### 3.2. Fatty Acid Status at Birth and Development of Allergy in Childhood

Fatty acid status at birth was compared between children who developed and did not develop allergy in seven studies [77,81,82,84,86,87,88](Table 2). EPA values at birth were significantly lower in children who developed allergy in three studies [77,82,86] that addressed the ages of birth to 2 years [77], 3 years in two studies [82,86], and 8 years [82]. Significantly higher AA/EPA ratio was reported for the ages of birth to 2 years in children who developed allergy in one study [77]. In apparent contrast, significantly lower dihomo-gamma-linolenic acid (C20:3-n-6) and AA values were reported in one study [88] for children who were diagnosed with allergy at the age of 12 months; however, neither EPA nor DHA values were reported in this study.

### 3.3. Relationship of Fatty Acid Status at Birth to Odds Ratios and Relative Risks of Allergy in Children

The wide variety of parameters characterizing fatty acid status and the widely different time points of evaluating allergy rendered it impossible to carry out formal meta-analysis within the present systematic review. However, odds ratios (ORs) or RRs of allergy reported in the studies reviewed allow some comparative mathematical representation of the results (Table 3). Altogether, 10 significantly different ORs or RRs for allergy were reported from five studies [76,82,84,85,86]. In four studies including altogether 1042 participants, n-3 LCPUFA supplementation during pregnancy [76] as well as high DHA or n-3 LCPUFA [84] or high eicosapentaenoic acid (EPA, C20:5n-3) in cord plasma [82,86] were associated with significantly reduced RRs or ORs of various clinical manifestations of allergy (Table 3). The extent of the reduction in ORs or RRs for allergy in favor of n-3 fatty acids was at least one third (highest OR or RR reported: 0.62). In a sub-study with 1191 participants of the Avon Longitudinal Study of Parents and Children [85], high RBC AA/EPA ratios were associated with enhanced OR of eczema at the age of 18 to 30 months, high AA/ALA ratios with enhanced OR of wheezing at the age of 30 to 42 months, whereas high ALA/n-3 LCPUFA ratios were marginally, but significantly associated with decreased OR of wheezing (Table 3).

### 3.4. Correlation between Fatty Acids and Allergy-Related Laboratory Parameters at Birth

Statistically significant correlations were reported between cord blood fatty acid values and some laboratory parameters considered by the authors of the given papers to be immunologically relevant (F_2_-isoprostanes, soluble CD23 receptors, interleukin (IL) 1β, 4-hydroxy-DHA, 14-hydroxy-DHA, 17-hydroxy-DHA, and 18-hydroxy-EPA) in five studies [75,78,79,80,86] (Table 4). In a study on 83 pregnant atopic women, cord red blood cell (RBC) EPA values were significantly and inversely associated to both cord blood plasma and urinary F_2_-isoprostanes, whereas cord blood RBC DHA values were significantly and inversely correlated with urinary F_2_-isoprostanes only [75]. Cord blood plasma EPA, docosapentaenoic acid (DPA), DHA, and total n-3 PUFA values were significantly and negatively correlated with soluble CD23 receptor levels in another study investigating 35 children who subsequently developed allergic sensitization and atopic dermatitis before the age of 3 years [86].

Cord blood plasma DHA values were significantly and inversely correlated to IL 1β concentrations in a study on 118 women participating in a n-3 LCPUFA supplementation trial [79]. In contrast, pooled maternal and cord plasma DHA values significantly and positively correlated with 4-hydroxy-DHA, 14-hydroxy-DHA, 17-hydroxy-DHA, and 18-hydroxy-EPA in a n-3 LCPUFA supplementation study in 60 pregnant women [78], whereas cord RBC EPA values significantly and positively correlated with 18-hydroxy-EPA values in another supplementation study including 83 participants [80] (Table 4).

## 4. Discussion

Recent developments in infant nutrition put further emphasis on the question of the role of LCPUFA supply to the fetus. Both a Cochrane Database Systematic Review on LCPUFA supplementation trials in infancy [89] and a systematic review and meta-analysis of fatty acid compositional data of human milk in various populations [90] substantially underpinned the importance of preformed DHA in infant nutrition. Today, DHA is a mandatory constituent of infant formula within the European Union [91], and mandatory inclusion of also AA is under vivid discussion in the medical literature [92,93]. If the diet of infants must contain preformed LCPUFA, it is logical to consider recommending for pregnant women the intake of certain amounts of certain type(s) of EFAs and/or LCPUFAs. Before embarking on recommendations, however, it is essential to better understand the potentially different effects of different LCPUFAs on various aspects of fetal development.

The major finding of the present review is the clear preventive relation between higher n-3 LCPUFA status at birth and the occurrence of allergy in childhood. When the dichotomous parameter of suffering or not from allergic condition in childhood was analyzed, EPA proved to be distinctive fatty acid in cord blood lipids. When the linear parameters of ORs and RRs were taken into consideration, besides EPA, DHA and total n-3 LCPUFA values were also significantly associated to clinically relevant reduction (more than 33%) in allergy. At birth, i.e., within the cord blood sample itself, higher EPA, docosapentaenoic acid, and DHA values were significantly and negatively associated with various laboratory parameters considered to reflect allergic trait.

Although it is generally accepted that DHA is the paramount LCPUFA in infant nutrition, based on the data obtained in the present systematic review it might be tempting to speculate that for the nutrition of the fetus, EPA may play an equally important role, at least as far as the intrauterine programming of the immune system is concerned. EPA will be converted by the cyclo-oxygenase and lipoxygenase enzymes into three and five series of prostaglandins and leukotrienes, which have less inflammatory effects than the two and four series of eicosanoids synthesized by the same enzymes from AA [94]. Furthermore, EPA serves as precursor for resolvins and maresins with anti-inflammatory effects [95]. However, data from animal studies indicate that tissue accretion of EPA is several folds lower than that of DHA or AA [96]. Therefore, isolated or unbalanced administration of EPA during pregnancy would not fit to the physiological fetal fatty acid accretion profiles. The data generated in the present systematic review cannot be directly translated into dietary advice but can serve as background for optimizing the design of further supplementation studies.

Among the seven studies comparing fatty acid status at birth between allergic and non-allergic children in our present systematic review, significantly lower contributions of EPA to cord blood lipids were reported in parallel with lack of difference in DHA values in three studies [77,82,86], whereas significant difference in DHA was not reported in any of the studies. However, the mostly observatory nature of the data reviewed here allows more to generate ideas than to support them.

It is to be noted that the appearance of allergic or atopic symptoms in the offspring may be differently related to LCPUFA status at birth in different groups of mothers. In the children of mothers with no allergy, LCPUFA supplementation in infancy significantly reduced the hazard ratios of any allergic diseases and skin allergic diseases, but failed to influence the hazard ratio of wheeze/asthma [41]. In the same study, in children of mothers with allergy, LCPUFA supplementation in infancy reduced the hazard ratio of wheeze/asthma but failed to influence the hazard for all allergic illnesses and skin allergic illness [41]. Moreover, population-specific associations were reported between blood parameters and asthma subtypes in a study involving altogether 3738 African American, Mexican American, and Puerto Rican children with median ages of 12 to 14 years [97].

Our present approach to try to differentiate between the effects of different n-3 LCPUFAs does not stand in the literature. Effects on n-3 LCPUFA supplementation either in the form of DHA or in the form of EPA were compared both in RCTs and in meta-analyses. In a randomized, crossover, head-to-head study in 154 healthy women and men with abdominal obesity and low-grade systematic inflammation, supplementation with DHA compared with supplementation with EPA resulted in significantly greater reduction in IL-18 values (DHA versus control: −18.15 ± 6.25 pmol/L, EPA versus control: −2.12 ± 6.29 pmol/L, mean ± SEM, DHA versus EPA: *p* = 0.01), whereas changes in C-reactive protein, IL-6, and tumor necrosis factor-α (TNF-α) were not significantly different [98]. The greater effect of DHA than that of EPA is in line with a meta-analysis showing that a major part of anti-inflammatory effects of marine-derived mixed n-3 LCPUFA can be attributed to DHA [99]. Gene expression analyses in 44 participants of the above-mentioned supplementation study showed no difference between EPA or DHA treatment on the expression of IL-10, IL-1β, and TNF-α genes [100]. Comparison in a 6-week trial of two different doses of EPA (600 mg/day or 1800 mg/day) with DHA (600 mg/day) in 121 healthy subjects showed no difference in IL-6, TNF-α, and vascular cell adhesion molecule 1 values among the three supplementation groups [101]. The anti-inflammatory effects of DHA and EPA were compared also in a recent pairwise and network meta-analyses of 5 and 20 RCTs including data from 411 and 1231 participants, respectively [102]. In both pairwise and network meta-analyses of these supplementation trials, EPA and DHA had similar effects on plasma C-reactive protein, IL-6, and TNF-α concentrations [102].

Growing interest and accumulating evidence in the immunological role of intrauterine LCPUFA status gave rise to systematic overviews of the effect of n-3 LCPUFA supplementation on childhood allergy as well [103,104]. Systematic review of five RCTs with altogether 949 participants showed that n-3 LCPUFA supplementation during pregnancy reduced 12-month prevalence of positive egg skin prick test (OR: 0.33; 95% CI: 0.16 to 0.70) and childhood asthma (OR: 0.35; 95% CI: 0.15 to 0.79), and significantly reduced cord blood IL-13 levels [103]. However, in two of the five studies reviewed by Klemens et al. [103], intrauterine LCPUFA supplementation was followed by similar supplementation during lactation, thus the findings reported cannot be attributed solely to the intervention during pregnancy. Five years later, another systematic review identified five prenatal and one prenatal/postnatal RCTs (including four out of the five in the review of Klemens et al. [103]) addressing the question of LCPUFA supplementation in pregnant women on allergy outcomes in their children [104]. N-3 LCPUFA (EPA and DHA) supplementation showed a clear reduction in medically diagnosed IgE mediated allergy in children aged 12 to 36 months (RR: 0.66; 95% CI: 0.44 to 0.98; 2 RCTs; 823 participants), but not beyond 36 months, or if allergy diagnoses on the basis of parental reports were included [104]. Seven RCTs involving 2047 children were included into a more recent systematic review and meta-analysis on the effects of n-3 LCPUFA supplementation during pregnancy on asthma or wheeze of children [105]. N-3 LCPUFA supplementation reduced significantly the incidence of wheeze/asthma (RR: 0.81; 95% CI: 0.66 to 0.99), while the incidence of childhood asthma was not significantly reduced (RR: 0.89; 95% CI: 0.67 to 1.17) [105].The above-mentioned systematic reviews [103,104] were aimed to obtain data on the clinical efficacy of n-3 LCPUFA supplementation during pregnancy, thus they included RCTs only. In the present systematic review, we addressed the question of the relationship of cord blood fatty acid status at birth (as surrogate cumulative parameter of fetal fatty acid status) to the development of allergy in childhood, thus we included all types of studies. Furthermore, characterization of LCPUFA status at birth was among the eligibility criteria in the present study, whereas it was no prerequisite in the previous systematic reviews. Consequently, from the 14 studies included into the present review, only one [77] was included into the previous reviews.

The present systematic review has various limitations which usually accompany studies relating fatty acid status to clinical diagnoses and/or to surrogate biochemical parameters of clinical outcomes. First, chromatographic methods of fatty acid determination are far from being standardized; there are differences in the type of sample analyzed, in the palette of the fatty acids reported, and in the way the results are expressed. Second, identical diagnostic expressions, such as “allergy” or “atopy”, might cover slightly different meanings in different studies. Third, laboratory parameters considered immune-related or allergy-related by the authors of the studies reviewed here may characterize fairly different degrees as well as different aspects of the development of allergy. Although our supplementary tables showing detailed description of the methods of fatty acid analyses as well as the rationality behind using a given laboratory parameter as immune- or allergy-related may underpin the reliability of our conclusion, there remains some methodological uncertainties that should be considered at the evaluation of the data reported.

EFA and LCPUFA intake of pregnant women is a practical question. Existing evidence suggest that some enhancement of EFA and LCPUFA intake of pregnant women may beneficially influence some pregnancy outcomes. The findings of our present systematic review may provide useful information for defining optimal EFA and LCPUFA intakes for pregnant women.

## Figures and Tables

**Table 2:** Comparison of fatty acid status at birth between allergic and non-allergic children.

First Author, Year of Publ.	Age at Allergy Investigation	Type of Sample	Type of Allergy	Fatty Acids in Allergic Patients
Yu G, 1996 [87]	in the first 6 years	Cord blood serum PL	Allergic disease	LA, DHGLA, AA: →; EPA, DPA, DHA: →
Byberg K, 2008 [86]	3 years	Cord blood plasma	Atopy	LA, GLA, DHGLA, AA: →; EPA: ↓; ALA, DHA: →
Montes R, 2013 [84]	14 months	Cord blood plasma	Atopy	LA, GLA, DHGLA, AA: →; ALA, EPA, DPA, DHA: →
Barman M, 2019 [81]	36 months	Cord blood serum PL	Allergy	AA: →
Barman M, 2020 [82]	3 years	Cord blood serum PL	Allergy	EPA: ↓; ALA, DPA, DHA: →; LA, DHGLA, AA: →
	8 years			EPA: ↓; ALA, DPA, DHA: →; LA, DHGLA, AA: →
Galli E, 1994 [88]	12 months	Cord blood serum PL	Atopy	LA: →; DHGLA, AA: ↓
Furuhjelm C, 2011 [77]	0–24 months	Cord blood plasma PL	>1 Allergic symptoms	EPA: ↓; DHA: →; AA/EPA: ↑

AA: arachidonic acid, ALA: alpha-linolenic acid, DHA: docosahexaenoic acid, DHGLA: Dihomo-gamma-linolenic acid, DPA: docosapentaenoic acid, EPA: eicosapentaenoic acid, GLA: gamma-linolenic acid, LA: linoleic acid, PL: phospholipids, ↑: significantly higher values in allergic patients, →: no significant difference between allergic and non-allergic patients, ↓: significantly lower values in allergic patients.

**Table 3:** Significantly different odds ratios/relative risks in the included studies.

First Author, Year of Publ.	Group	Age at Investigation	Type of Allergy	RR/OR
Best KP, 2018 [76]	n-3 LCPUFA supplementation	1 years	Wheeze symptoms with sensitization	adjusted RR: 0.52 (*p* = 0.03)
		1 years	Egg sensitization	adjusted RR: 0.62 (*p* = 0.02)
		6 years	D. farinae sensitization	adjusted RR: 0.62 (*p* = 0.02)
Barman M, 2020 [82]	Cord blood EPA	3 years	Allergy	OR: 0.20 (*p* = 0.045)
Byberg K, 2008 [86]	High EPA in cord blood (upper quartile)	3 years	Atopy	RR: 0.3 (*p* = 0.03)
Montes R, 2013 [84]	Cord plasma DHA	6 and 14 months	Eczema	adjusted OR: 0.50 (*p* = 0.01)
	Cord plasma n-3 LCPUFA			adjusted OR: 0.49 (*p* = 0.00)
Newson RB, 2004 [85]	Cord RBC AA/EPA	18–30 months	Eczema	adjusted OR: 1.14 (*p* = 0.044)
	Cord RBC LA/ALA	30–42 months	Wheezing	adjusted OR: 1.04 (*p* = 0.019)
	Cord RBC ALA/n-3			adjusted OR: 0.98 (*p* = 0.040)

AA: arachidonic acid, ALA: alpha-linolenic acid, DHA: docosahexaenoic acid, EPA: eicosapentaenoic acid, LA: linoleic acid, LCPUFA: long-chain polyunsaturated fatty acid, OR: odds ratio, RBC: red blood cells, RR: relative risk.

**Table 4:** Significant correlations between fatty acids and allergy-related laboratory parameters at birth.

First Author, Year of Publ.	Age at Investigation	Fatty Acid	Dependent Variable	β or r (*p*)
Barden AE, 2004 [75]	Birth	Cord RBC EPA	Cord plasma F_2_-isoprostanes	r = −0.351 (*p* = 0.001)
		Cord RBC EPA	Urinary F_2_-isoprostanes	r = −0.290 (*p* = 0.017)
		Cord RBC DHA		r = −0.241 (*p* = 0.05)
Byberg K, 2008 [86]	Birth	n-3 PUFA	sCD23	r = −0.28 (*p* = 0.018)
		DHA		r = −0.26 (*p* = 0.031)
		EPA		r = −0.26 (*p* = 0.03)
		DPA		r = −0.2 (*p* = 0.026)
Mozurkewich EL, 2018 [79]	Birth	Cord blood DHA	IL 1β	Neg. corr. (*p* = 0.03)
Mozurkewich EL, 2016 [78]	Birth	Log cord plasma DHA *	log 4-HDHA	r = 0.51 (*p* < 0.001)
			log 14-HDHA	r = 0.47 (*p* < 0.001)
			log 17-HDHA	r = 0.34 (*p* < 0.02)
See, 2017 [80]	Birth	Cord RBC EPA	18-HEPE	B = 151.4 (*p* < 0.001)

DHA: docosahexaenoic acid, DPA: docosapentaenoic acid, EPA: eicosapentaenoic acid, 4-HDHA: 4-hydroxy-docosahexaenoic acid, 14-HDHA:14-hydroxy-docosahexaenoic acid, 17-HDHA: 17-hydroxy-docosahexaenoic acid, 18-HEPE: 18-hydroxy-eicosapentaenoic acid, log: logarithm-transformed values, PUFA: polyunsaturated fatty acid, RBC: red blood cells, sCD23: soluble CD23 receptor, *: maternal and cord plasma samples pooled together.

## Data Availability

Not applicable.

## Supplementary Materials

The following supporting information can be downloaded at: https://www.mdpi.com/article/10.3390/life12040526/s1, Table S1: Prisma 2020 Checklist. List S1: Search strategy for Ovid MEDLINE. Table S2: Determined fatty acids and methods of determination in the included studies. Table S3: Immune-related biochemical factors and their usefulness in allergic diseases. Table S4: Risk of bias assessment for included non-randomized (cohort and case-control). Table S5: Risk of bias assessment for included randomized controlled trials.
