# Supplementary material for: Long-Chain Polyunsaturated Fatty Acid Status at Birth and Development of Childhood Allergy: A Systematic Review
Source: Life (Basel). 2022 Apr 2;12(4):526. doi: 10.3390/life12040526 (PMC9030843; doi:10.3390/life12040526)
Supplement: Supplementary file 1 [file life-12-00526-s001.zip › life-1602015-SI.pdf]

life

MDPI

Supplementary Material Long-Chain Polyunsaturated Fatty Acid Status at Birth and Development of Childhood Allergy: A Systematic Review

Table S1. Prisma 2020 Checklist.

Section and Topic	Item #	Checklist Item	Location Where Item is Reported
		**Title**	
Title	1	Identify the report as a systematic review.	Page 1
		**Abstract**	
Abstract	2	See the PRISMA 2020 for Abstracts checklist.	Page 1
		**Introduction**	
Rationale	3	Describe the rationale for the review in the context of existing knowledge.	Page 2
Objectives	4	Provide an explicit statement of the objective(s) or question(s) the review addresses.	Page 2
		**Methods**	
Eligibility criteria	5	Specify the inclusion and exclusion criteria for the review and how studies were grouped for the syntheses.	Page 2-3
Information sources	6	Specify all databases, registers, websites, organisations, reference lists and other sources searched or consulted to identify studies. Specify the date when each source was last searched or consulted.	Page 3
Search strategy	7	Present the full search strategies for all databases, registers and websites, including any filters and limits used.	Page 3; Suppl. List 1
Selection process	8	Specify the methods used to decide whether a study met the inclusion criteria of the review, including how many reviewers screened each record and each report retrieved, whether they worked independently, and if applicable, details of automation tools used in the process.	Page 3
Data collection process	9	Specify the methods used to collect data from reports, including how many reviewers collected data from each report, whether they worked independently, any processes for obtaining or confirming data from study investigators, and if applicable, details of automation tools used in the process.	Page 3
Data items	10a	List and define all outcomes for which data were sought. Specify whether all results that were compatible with each outcome domain in each study were sought (e.g. for all measures, time points, analyses), and if not, the methods used to decide which results to collect.	Page 2
	10b	List and define all other variables for which data were sought (e.g. participant and intervention characteristics, funding sources). Describe any assumptions made about any missing or unclear information.	Page 3
Study risk of bias assessment	11	Specify the methods used to assess risk of bias in the included studies, including details of the tool(s) used, how many reviewers assessed each study and whether they worked independently, and if applicable, details of automation tools used in the process.	Page 3
Effect measures	12	Specify for each outcome the effect measure(s) (e.g. risk ratio, mean difference) used in the synthesis or presentation of results.	n.a.
Synthesis methods	13a	Describe the processes used to decide which studies were eligible for each synthesis (e.g. tabulating the study intervention characteristics and comparing against the planned groups for each synthesis (item #5)).	n.a.
	13b	Describe any methods required to prepare the data for presentation or synthesis, such as handling of missing summary statistics, or data conversions.	n.a.
	13c	Describe any methods used to tabulate or visually display results of individual studies and syntheses.	n.a.
	13d	Describe any methods used to synthesize results and provide a rationale for the choice(s). If meta-analysis was performed, describe the model(s), method(s) to identify the presence and extent of statistical heterogeneity, and software package(s) used.	n.a.

Section and Topic	Item #	Checklist Item	Location Where Item is Reported
	13e	Describe any methods used to explore possible causes of heterogeneity among study results (e.g. subgroup analysis, meta-regression).	n.a.
	13f	Describe any sensitivity analyses conducted to assess robustness of the synthesized results.	n.a.
Reporting bias assessment	14	Describe any methods used to assess risk of bias due to missing results in a synthesis (arising from reporting biases).	n.a.
Certainty assessment	15	Describe any methods used to assess certainty (or confidence) in the body of evidence for an outcome.	n.a.
		**Results**	
Study selection	16a	Describe the results of the search and selection process, from the number of records identified in the search to the number of studies included in the review, ideally using a flow diagram.	Figure 1 (page 4); Page 3-4
	16b	Cite studies that might appear to meet the inclusion criteria, but which were excluded, and explain why they were excluded.	Page 3-4
Study characteristics	17	Cite each included study and present its characteristics.	Table 1 (page 5-6)
Risk of bias in studies	18	Present assessments of risk of bias for each included study.	Page 4; Table S4-5
Results of individual studies	19	For all outcomes, present, for each study: (a) summary statistics for each group (where appropriate) and (b) an effect estimate and its precision (e.g. confidence/credible interval), ideally using structured tables or plots.	Table 2, 3, 4
Results of syntheses	20a	For each synthesis, briefly summarise the characteristics and risk of bias among contributing studies.	Page 6-7
	20b	Present results of all statistical syntheses conducted. If meta-analysis was done, present for each the summary estimate and its precision (e.g. confidence/credible interval) and measures of statistical heterogeneity. If comparing groups, describe the direction of the effect.	n.a.
	20c	Present results of all investigations of possible causes of heterogeneity among study results.	n.a.
	20d	Present results of all sensitivity analyses conducted to assess the robustness of the synthesized results.	n.a.
Reporting biases	21	Present assessments of risk of bias due to missing results (arising from reporting biases) for each synthesis assessed.	n.a.
Certainty of evidence	22	Present assessments of certainty (or confidence) in the body of evidence for each outcome assessed.	n.a.
		**Discussion**	
Discussion	23a	Provide a general interpretation of the results in the context of other evidence.	Page 8-9
	23b	Discuss any limitations of the evidence included in the review.	Page 9
	23c	Discuss any limitations of the review processes used.	Page 9
	23d	Discuss implications of the results for practice, policy, and future research.	Page 10
		**Other Information**	
Registration and protocol	24a	Provide registration information for the review, including register name and registration number, or state that the review was not registered.	Page 2
	24b	Indicate where the review protocol can be accessed, or state that a protocol was not prepared.	Page 3
	24c	Describe and explain any amendments to information provided at registration or in the protocol.	Page 3
Support	25	Describe sources of financial or non-financial support for the review, and the role of the funders or sponsors in the review.	Page 10
Competing interests	26	Declare any competing interests of review authors.	Page 10
Availability of data, code and other materials	27	Report which of the following are publicly available and where they can be found: template data collection forms; data extracted from included studies; data used for all analyses; analytic code; any other materials used in the review.	Page 10

From: Page MJ, McKenzie JE, Bossuyt PM, Boutron I, Hoffmann TC, Mulrow CD, et al. The PRISMA 2020 statement: an updated guideline for reporting systematic reviews. BMJ 2021;372:n71. doi: 10.1136/bmj.n71. For more information, visit: http://www.prisma-statement.org/ (Access date: 20.02.2022)

List S1. Search strategy for Ovid MEDLINE.

1. Cord.mp
2. Newborn.mp
3. Infant.mp
4. Perinatal.mp
5. Postnatal.mp
6. 1 OR 2 OR 3 OR 4 OR 5
7. Immune.mp
8. Immun*.mp
9. Allergy.mp
10. Allerg*.mp
11. Atopy.mp
12. Atopic.mp
13. Inflammation.mp
14. Infection.mp
15. 7 OR 8 OR 9 OR 10 OR 11 OR 12 OR 13 OR 14
16. Arachidonic.mp
17. Arachidonic acid.mp
18. Docosahexaenoic.mp
19. Docosahexenoic.mp
20. Docosahexaenoic acid.mp
21. 16 OR 17 OR 18 OR 19 OR 20
22. 6 AND 15 AND 21
23. Limit 22 to animals
24. 22 NOT 23

Table S2. Determined fatty acids and methods of determination in the included studies.

First Author, Year of Publication	Method of Fatty Acid Determination (Column)	Published Fatty Acids (Determined Fatty Acids) [Concentration Unit]
Barden AE, 2004 [68]	GC	C20:4n-6, C20:5n-3, C22:6n-3 [μg/10^6 cells]
Barman M, 2019 [74]	GC-FID (SPB-5, 30 m×0.25 mm×0.25 μm and DB-WAX 30 m×0.25 mm×0.25 μm)	C18:0, C18:1n-7, C18:1n-9, C18:2n-6, C18:3n-3, C20:0, C20:3n-6, C20:4n-6, C20:5n-3, C22:0, C22:4n-6, C22:5n-6, C22:5n-3, C22:6n-3 [w/wt%]
Barman M, 2020 [75]	GC-MS (VF-WAXms, 30 m×0.25 mm×0.25 μm)	C14:0, C16:0, C16:1-7, C18:0, C18:1n-9, C18:2n-6, C18:3n-3, C20:0, C20:3n-6, C20:4n-6, C20:5n-3, C22:0, C22:4n-6, C22:5n-3, C22:5n-6, C22:6n-3, C24:0 (total n=18 FAs) [w/wt%]
Best KP, 2018 [69]	Capillary GC	ω-3 LCPUFA [w/wt%]
Byberg K, 2008 [79]	GC-FID, Varian CP-Wax52 CB 25 m×0.25 mm×0.2 μm	C20:1n-9, C18:2n-6, C18:3n-6, C20:3n-6, C20:4n-6, C18:3n-3, C20:5n-3, C22:6n-3 (total n=26 FAs) [μmol/l]
Dirix CEH, 2009 [76]	GC	C18:3n-6, C20:4n-6, C20:5n-3, C22:6n-3 [w/wt%]
Furuhjelm C, 2011 [70]	GC-FID	C18:2n-6, C20:4n-6, C18:3n-3, C20:5n-3, C22:6n-3 [mol%]
Galli E, 1994 [81]	GC-MS (HP FFA-P; 60 m×0.32mm)	C18:2n-6, C18:3n-6, C20:4n-6 [w/wt%]
Montes R, 2013 [77]	GC-FID (Varian VF-23ms, 10 m×0.10 mm ×0.10 μm)	C14:0, C15:0, C16:0, C17:0, C18:0, C16:1n-7, C18:1n-9/n7, C20:1n-9, C24:1, C18:2n-6, C18:3n-6, C18:3n-3, C20:2n-6, C20:3n-9, C20:3n-6, C20:4n-6, C20:5n-3, C22:2n-6, C22:4n-6, C22:5n-6, C22:5n-3, C22:6n-3 [w/wt%]
Mozurkewich EL, 2018 [72]	GC-MS	C22:6n-3 [w/w%]
Newson RB, 2004 [78]	GC-FID	C18:3n-3, C18:4n-3, C20:5n-3, C22:5n-3, C22:6n-3, C18:2n-6, C18:3n-6, C20:3n-6, C20:4n-6, C22:4n-6, C22:5n-6; (total n=40 FAs) [w/wt%]
See VHL, 2017 [73]	GC	C20:5n-3, C22:6n-3, C18:2n-6, C20:4n-6, [w/wt%]
Yu G, 1996 [80]	capillary GC	C18:2n-6, C20:2n-6, C20:3n-6, C20:4n-6, C22:4n-6, C20:5n-3, C22:5n-3, C22:6n-3; (C14-C22) [w/wt%]

FA: fatty acid, GC: gas chromatography, GC-FID: gas chromatography with flame ionization detector, GC-MS: gas chromatography with mass spectrometry, LCPUFA: long-chain polyunsaturated fatty acid.

Table S3. Immune-related biochemical factors and their usefulness in allergic diseases.

Immune-Related Factors	Usefulness in Alleric Diseases
F_2-isoprostanes	Prostaglandin-like compounds generated after oxidative stress; marker of oxidative stress
Soluble CD23 (sCD23)	low affinity IgE receptor (indicates IgE metabolism)
Interleukin 1β	key mediator of the inflammatory response (pathophysiology of chronic inflammatory diseases e.g. asthma)
4-hydroxy-DHA (4-HDHA)	Pathway marker for the D-series resolvins 5-lipoxygenase; antiangiogenic effect
14-hydroxy-DHA (14-HDHA)	Pathway marker for maresins; 12-lipoxygenase; stimulate macrophage phenotype switch from M1 to M2, enhance phagocytosis and bacterial killing
17-hydroxy-DHA (17-HDHA)	Pathway marker for the D-series resolvins / protectins (high expression in eosinophils and macrophages); 15-lipoxygenase; promote inflammation resolution
18-hydroxy-EPA (18-HEPE)	EPA-derived resolvin (E-series resolvin); 5-lipoxygenase; promote inflammation resolution

Table S4. Risk of bias assessment for included non-randomized (cohort and case-control) studies.

Study ID	Bias due to Confounding	Selection of Participants	Classification of Interventions	Deviation from Intended Interventions	Missing Data	Measurement of Outcomes	Selection of Reported Results	Overall Risk of Bias
Barman M, 2019	Low	Moderate	Low	Low	Moderate	Low	Moderate	Moderate
Barman M, 2020	Low	Moderate	Low	Low	Moderate	Low	Moderate	Moderate
Dirix CEH, 2009	Low	Moderate	Low	Low	Low	Low	Moderate	Moderate
Galli E, 1994	Low	Serious	Low	Low	Low	Low	Moderate	Serious
Montes R, 2013	Low	Moderate	Low	Low	Low	Low	Moderate	Moderate
Newson RB, 2004	Moderate	Moderate	Moderate	Low	Moderate	Low	Serious	Serious
Byberg K, 2008	Moderate	Moderate	Low	Low	Low	Low	Moderate	Moderate
Yu G, 1996	Low	Moderate	Low	Low	Low	Low	Moderate	Moderate

Table S5. Risk of bias assessment for included randomized controlled trials.

Study ID	Randomisation Process	Deviations from the Intended Interventions	Missing Outcome Data	Measurement of the Outcome	Selection of the Reported Results	Overall Risk of Bias
Barden AE, 2004	Low	Some concerns	Low	Some concerns	Some concerns	Some concerns
Best KP, 2018	Low	Low	Low	Low	Some concerns	Some concerns
Furuhjelm C, 2011	Some concerns	Low	Low	Low	Some concerns	Some concerns
Mozurkewich EL, 2016	Low	Low	Low	Low	Some concerns	Some concerns
Mozurkewich EL, 2018	Low	Low	Low	Low	Some concerns	Some concerns
See VHL, 2017	Low	Low	Low	Low	Some concerns	Some concerns
